# The Immunology of Wild Rodents: Current Status and Future Prospects

**DOI:** 10.3389/fimmu.2017.01481

**Published:** 2017-11-14

**Authors:** Mark Viney, Eleanor M. Riley

**Affiliations:** ^1^School of Biological Sciences, University of Bristol, Bristol, United Kingdom; ^2^Department of Immunology and Infection, London School of Hygiene and Tropical Medicine, London, United Kingdom

**Keywords:** mouse, rat, vole, rodent, immune, immunology, wild

## Abstract

Wild animals’ immune responses contribute to their evolutionary fitness. These responses are moulded by selection to be appropriate to the actual antigenic environment in which the animals live, but without imposing an excessive energetic demand which compromises other component of fitness. But, exactly what these responses are, and how they compare with those of laboratory animals, has been little studied. Here, we review the very small number of published studies of immune responses of wild rodents, finding general agreement that their humoral (antibody) responses are highly elevated when compared with those of laboratory animals, and that wild rodents’ cellular immune system reveals extensive antigenic exposure. In contrast, proliferative and cytokine responses of *ex vivo*-stimulated immune cells of wild rodents are typically depressed compared with those of laboratory animals. Collectively, these responses are appropriate to wild animals’ lives, because the elevated responses reflect the cumulative exposure to infection, while the depressed proliferative and cytokine responses are indicative of effective immune homeostasis that minimizes immunopathology. A more comprehensive understanding of the immune ecology of wild animals requires (i) understanding the antigenic load to which wild animals are exposed, and identification of any key antigens that mould the immune repertoire, (ii) identifying immunoregulatory processes of wild animals and the events that induce them, and (iii) understanding the actual resource state of wild animals, and the immunological consequences that flow from this. Together, by extending studies of wild rodents, particularly addressing these questions (while drawing on our immunological understanding of laboratory animals), we will be better able to understand how rodents’ immune responses contribute to their fitness in the wild.

## On Finding That Your Experimental Model is Wrong

Model experimental systems—*E. coli*, yeast, *Drosophila, C. elegans*, mice—are a bedrock of modern experimental biology. Enormous investment has been made in these models, and they underpin large, international research efforts. Choosing to work with the right model can make or break an academic career.

One of us (Mark Viney) has used a simple laboratory model—infecting laboratory rats (*Rattus norvegicus)* with the nematode parasite *Strongyloides ratti* (1–4). Moreover, the model had a purity: in the wild *S. ratti* infects *R. norvegicus*, so the model was simply moving parasites from wild rats into laboratory rats. Experiments using this elegantly simple model had discovered how the parasite’s life cycle was controlled by a range of environmental effects, including the host immune response (1). This was a fascinating discovery, illuminating an association of such intimacy, where a parasite used the host to control its life cycle decisions.

But there was a gnawing problem. Each rat that was infected became immune to *S. ratti* after about 5 weeks, expelling the worms and becoming resistant to reinfection (5). Because of this, parasites were maintained by continually infecting parasite-naïve rats. In contrast, in the wild, almost two-thirds of rats were infected with *S. ratti* (6), suggesting that wild animals were not becoming immune to *S. ratti*, as their lab cousins so readily were. If the immunology of lab rats infected with *S. ratti* was so different to that of wild rats, then what had this lab model really revealed about how the host immune response actually controlled the parasite’s life cycle in the wild? Intuitively, we reasoned that laboratory and wild rats were immunologically different: wild animals were leading stressful, resource-limited lives, and so were making low-level, insufficient immune responses against parasites, and that was why *S. ratti* infections were so common in the wild.

Intuition is one thing; what did the literature say? The answer—remarkably little. We could find almost no studies of the immune systems of wild rodents, and the little evidence that existed did not obviously support the hypothesis that wild rodents’ immune responses were impaired or impoverished. There were rather more studies of laboratory animals, livestock, as well as some wild animals (mainly birds), generally supporting the idea that immune resources were energetically and resource costly (7), and so one could argue that there were likely to be some resource-based constraints on wild rodents’ immune responses. But, overall, there was not any clear information on what immune responses wild rodents were making or how this might explain why *S. ratti* infections were so much more common in the wild than laboratory studies predicted that they would be. This disconnect from the lab to the wild in this hitherto, elegantly simple model—and that so little was known about wild animal immunology in general—spurred our determined look into wild rodents’ immune responses.

## Key Concepts in Eco-Immunology

Eco-immunology is the study of the immune responses of wild animals in ecologically relevant settings. The broader rationale for studying the immune responses of wild animals is that these responses contribute to wild animals’ evolutionary fitness (7). Immune systems respond to antigenic stimuli received by an animal and so different individuals within a population, different populations of a species, and different species, will each have qualitatively and quantitatively different exposure to antigens. Mammalian immune systems are adaptable and will respond, in one way or another, to any antigen they encounter. However, selection will act to optimize the form and nature of these immune responses to maximize fitness, in the context of other selection pressures to which animals are subject (8, 9). This leads to the first key concept of eco-immunology: immunoheterogeneity, so that different species, different populations within species, or different individuals within populations may differ in the immune responses that they make. These differences will be seen (i) in the resting status of the immune system, observed as the standing immune response, but also (ii) when animals are compared for their responses to a standard antigenic challenge, for example to vaccination. Animals will differ in these regards for both intrinsic reasons (e.g., genetically) and for extrinsic, contextual reasons, for example their different exposure to infection, and other challenges during their lives. It is appropriate that immunoheterogeneity is the first key concept of eco-immunology, because understanding both the ultimate and proximate causes of this heterogeneity, and its consequences, is arguably the central question in eco-immunology.

Making immune responses is just one aspect of an animal’s physiological demands. In addition, animals have to grow, seek, and compete for food and mates, and reproduce. All of these processes require energy. It is clear that immune responses are energetically demanding (7), as too is growing, foraging, and reproducing. Therefore, with the assumption that many wild animals are energy limited, then these limited resources have to be deployed among these competing physiological and life-history processes in such a way as to maximize evolutionary fitness. This means that the immune responses of wild animals may be sub-maximal because of energy limitation. This is the second key concept of eco-immunology: that individuals’ immune responses may be constrained by resource availability, and more generally that immune responses can be affected by an animal’s wider physiological state. Further, variation among individuals in the quantities of resource they have and in how they allocate these resources to immune function (or not) importantly contributes to immunoheterogeneity (key concept 1, above).

Animals are exposed to and infected with a myriad of organisms—viruses, bacteria, fungi, protozoa, and metazoa, both internally and externally (7). The vast majority of these are usually harmless commensals which the immune system has to learn to ignore, but a small proportion are potentially dangerous pathogens that need to be recognized and, if possible, eliminated. Being infected with these organisms is a normal part of animal life, and animals and their immune systems have evolved in their presence. All of these organisms are sources of antigenic stimulus for the immune system, though the nature and degree of this stimulation varies among different types of organisms, their number, and their location in, or on, their host. This is the third key concept of eco-immunology: that animals have a potentially very large and diverse antigenic load. This antigenic load will differ among individuals within populations, among populations within species, and among species, and so also contribute to immunoheterogeneity (key concept 1, above).

Applying these three key concepts to laboratory and wild animals reveals substantial differences between them. In laboratory animals, immunoheterogeneity (key concept 1) among individuals within a species is minimized, if not extinguished, while in wild animals it exists in abundance. Laboratory animals are rarely resource limited (key concept 2), suggesting that their immune responses are maximal, while in wild animals we presume that resource limitation is widespread and so that immune responses may be sub-maximal (though they may be optimal) (10). Laboratory animals are usually maintained with a much reduced burden of infection (key concept 3), while wild animals have infections in abundance. Therefore, fully understanding the immune responses of animals and how they contribute to their fitness absolutely requires that they are studied in wild populations—this is the *raison d’eˆtre* of eco-immunology. However, the enormously detailed understanding of mammalian immune systems that has come from the extensive study of laboratory animals will play a central role in this endeavour, giving eco-immunologists both the tools to probe the immune systems of wild animals and some founding concepts from which to interpret data from these studies.

## Immune Function in Wild Rodents

There are notably few studies of the immune systems of wild rodents, whereas immunological studies of laboratory rodents abound. Specifically, a Web of Knowledge search for publications whose titles and abstracts mention “mouse/mice” and “immun-” finds 19,997 papers in 2016 alone (and 187,154 between 2006 and 2016, inclusive). By contrast, we are aware of only 26 published studies of the immunology of wild rodents. A predominance *per se* of studies of laboratory models over wild systems is not necessarily problematic but, as discussed above, extrapolation from laboratory models to wild animals is unlikely to be straightforward so that the underrepresentation of the immunological study of wild animals is worrying.

The published studies of wild rodent immunology are summarized in Table 1 (11–36). The criterion for inclusion was (i) that the animals had to directly originate from the wild (though we include studies that use pet shop-acquired animals too) and (ii) that some immunological parameter was measured. Here, we review these studies.

**Table 1:** A summary of studies of the immunology of wild rodents, (A) where wild and laboratory animals have been compared and (B) where wild animals only have been studied.

Author	Species	Sample Size	Reference
**(A)**			
Lochmiller et al. (1991)	*Mus musculus*	1 wild; 6 laboratory	(11)
Devalapalli et al. (2006)	Mice	10 wild; 24 laboratory	(12)
Abolins et al. (2011)	*Mus musculus domesticus*	33 wild; 32 laboratory	(13)
Boysen et al. (2011)	*M. musculus*	22 wild; 31 laboratory	(14)
Beura et al. (2016)	*M. musculus*	10 wild; 6 pet shop; 9 laboratory	(15)
Abolins et al. (2017)	*M. musculus domesticus*	460 wild; 181 wild compared with 64 laboratory	(16)
Japp et al. (2017)	*M. musculus*	Unspecified pet shop; unspecified laboratory	(17)
Lochmiller et al. (1993)	*Sigmodon hispidus*	47 wild; 67 captive	(18)
Devalapalli et al. (2006)	Rat	58 wild; 15 laboratory	(12)
Lesher et al. (2006)	*Rattus norvegicus*	54 wild; unspecified laboratory	(19)
Kataranovski et al. (2009)	*R. norvegicus*	48 wild; 48 laboratory	(20)
Kataranovski et al. (2009)	*R. norvegicus*	48 wild; 48 laboratory	(21)
Trama et al. (2012)	*R. norvegicus*	8 wild; 7 laboratory	(22)
Beldomenico et al. (2008)	*Microtus agrestis*	1,574 wild; 186 captive	(23)

**(B)**			
Lochmiller et al. (1992)	*S. hispidus*	108	(24)
Vestey et al. (1993)	*S. hispidus*	131 captive and wild caught	(25)
Lochmiller et al. (1994)	*S. hispidus*	310	(26)
Davis et al. (1979)	*R. norvegicus*	39	(27)
Shonnard et al. (1979)	*R. norvegicus*	48	(28)
Andrianaivoarimanana et al. (2012)	*Rattus rattus*	425	(29)
Beldomenico et al. (2008)	*M. agrestis*	771	(30)
Jackson et al. (2011)	*M. agrestis*	307	(31)
Beldomenico et al. (2008)	*M. agrestis*	1,574	(23)
Arriero et al. (2017)	*M. agrestis*	60	(32)
Sinclair and Lochmiller (2000)	*Microtus ochrogaster*	140	(33)
Lochmiller et al. (1991)	*Microtus pinetorum*	7	(11)
Lochmiller et al. (1991)	*Peromyscus leucopus*	29	(11)
Schwanz et al. (2011)	*P. leucopus*	49	(34)
Lehmer et al. (2010)	*Peromyscus maniculatus*	633	(35)
Lochmiller et al. (1991)	*Neotoma floridana*	4	(11)
Lochmiller et al. (1991)	*Onychomys leucogaster*	2	(11)
Lochmiller et al. (1991)	*Perognathus hispidus*	7	(11)
Jackson et al. (2009)	*Apodemus sylvaticus*	100	(36)

### Comparisons of Wild and Laboratory Rodent Immune Responses

There are seven published studies of mice, *Mus musculus*, that explicitly compared wild and laboratory animals. Two of these studies were experimental, where wild-caught mice were immunized—either with sheep red blood cells (SRBC) or with keyhole limpet haemocyanin (KLH)—and the effect compared with the same immunization of laboratory mice (11, 13). In both cases, the immune responses of the wild mice were greater than those of laboratory mice, seen as higher anti-KLH antibody titres and greater antibody avidity (13) and greater SRBC lytic effect (11).

The remaining five studies of mice were observational, and measured and compared, various immune parameters between wild and laboratory mice. Comparisons of spleen cell populations in wild and laboratory *M. musculus* (13, 14) showed that wild mice had proportionately more CD4^+^ T cells and greater numbers of activated CD4^+^ T cells than laboratory mice and that their B cells, dendritic cells (DCs), macrophages, and natural killer (NK) cells had a more activated phenotype. However, spleen cells from wild and laboratory mice produced similar amounts of interferon-γ (IFN-γ) after *in vitro* restimulation with the T cell mitogen concanavalin A (Con A) (13). In a separate study, *in vitro* restimulation of splenocytes with exogenous cytokines resulted in more rapid expression of the high affinity IL-2 receptor (CD25) by NK cells of wild mice compared to laboratory mice and these cells were more likely to produce IFN-γ (14). A more detailed comparison of wild, pet shop-derived mice (in some way a halfway-house between wild and laboratory animals) and laboratory mice (15) showed that the non-laboratory mice had higher frequencies of antigen-experienced, terminal effector and tissue-resident CD8^+^ cells compared with laboratory mice (15).

In our own recent work with wild *Mus musculus domesticus*, we undertook a systematic, immune-system wide analysis using a large sample of mice from different populations across the southern UK (16). This analysis also found that, by many measures, the immune systems of wild mice were more activated, or more antigen-experienced, than those of laboratory mice. This was seen as significantly higher concentrations of serum immunoglobulins (IgG, IgE) and acute phase proteins (serum amyloid P and haptoglobin), and as the wild mice splenocytes having proportionately more T cells, higher T cell: B cell and higher CD4^+^:CD8^+^ cell ratios, and more CD11b^+^ myeloid cells (16). These data were consistent with earlier work which found wild mice to have higher IgG and IgE titres than laboratory mice, although no difference in IgM titres (12). Significantly, the status that the CD4^+^ and CD8^+^ cells were markedly different between wild and laboratory mice, with wild mouse cells being comparatively more likely to be effector or effector memory cells than naïve cells, while the opposite was the case for the laboratory mice (16). There was very marked interindividual heterogeneity in almost all immune measures of wild mice, which was much more extensive than among the laboratory mice (16). Wild mouse NK cells were also found to be in a comparatively highly activated state (16), also supporting previous observations (14). Among myeloid cells, wild mice were found to have a sub-population of these cells—which we have called hypergranulocytic myeloid cells—that appear not to have been described from laboratory mice (16).

In notable contrast to these many signatures of activation of the immune responses in wild mice, the production of nine cytokines (IFN-γ, IL-1β, IL-4, IL-6, IL-10, IL-12p40, IL-12p70, IL-13, and MIP-2α) from wild mouse splenocytes following *in vitro* stimulation with four pathogen-associated ligands tended to be much lower than from cells of laboratory mice (16). Specifically, among 45 comparisons (5 culture conditions and 9 cytokines) there were 16 significant differences in cytokine concentrations between wild and laboratory mice, of which 13 were lower in the wild mice, compared with the laboratory mice. The only exception to this trend was that some cytokine responses (IFN-γ, IL-4, and MIP-2α) to a T cell mitogen were significantly higher among wild than lab mice (16). One interpretation of these data is that in wild mice there are substantial antigen-specific responses, but innate immune responses to pathogen ligands are highly constrained (possibly a homeostatic mechanism to prevent overwhelming inflammation in the face of continued pathogen challenge).

Detailed cytometric comparison of pet shop mice with laboratory mice [specific pathogen free (SPF), non-SPF, or quarantine mice] (17) showed differences among the groups, with pet shop mice having higher frequencies of innate immune cells, particularly NK cells (17), again a finding consistent with other studies (14). Pet shop mice also had notably more granulocytes, monocytes, and DCs; higher frequencies of antigen-experienced B cells and plasma cells; more effector and memory CD4^+^ and CD8^+^ cells, all consistent with their likely greater exposure to infection when compared with the laboratory mice (17). After polyclonal stimulation (with phorbol-myristate-acetate and ionomycin), pet shop mouse splenic CD4^+^ and CD8^+^ T cells produced more IFN-γ and IL-17A than the laboratory mice, but less TNF-α, among seven cytokines assayed in total (17).

Collectively, these seven studies of *Mus* show a broadly consistent picture: that the immune systems of wild mice are more activated and show evidence of greater antigen exposure (higher antibody titres, greater antibody avidity, proportionally more effector cells, and a higher activation state of those cells) than the immune systems of laboratory mice. This is commensurate with the *a priori* expectation that wild mice, presumably, are continually and repeatedly exposed to a diverse repertoire of commensal and pathogenic organisms. Interestingly, co-housing of laboratory mice with pet shop mice results in a rather rapid expansion of antigen-experienced (CD44^hi^) CD8^+^ T cells in laboratory mice, suggesting indeed that exposure of laboratory mice to infections from wild mice drives the immune system toward the immunological phenotype of wild mice (15). Somewhat surprisingly, there is a consistent finding that innate immune function (as measured by cytokine production) tends to be depressed in wild mice compared to laboratory mice, whereas the antigen-experienced T cells of wild mice are activated.

Beyond mice, studies have also compared the immune systems of wild and laboratory rats, *R. norvegicus*. Overall, peripheral thymic and spleen cell populations of wild and laboratory rats tended to be rather similar, though wild rat spleens had proportionally fewer CD4^+^ T cells and more CD8^+^ T cells (22) than laboratory rats. Also, the proportion of peripheral T cells expressing CD62L was lower, and the proportion expressing major histocompatibility (MHC) Class II was higher, in wild rats compared to laboratory rats, consistent with higher proportions of antigen-experienced effector memory T cells among wild rats (22). However, the picture of T cell maturation was complicated, with wild rats having higher proportions of immature CD4^+^CD8^+^ double-positive peripheral T cells, but lower proportions of immature CD90^+^CD4^+^ T cells, and higher proportions of CD59^+^CD8^+^ cells (a marker involved in complement regulation) (22). Two other studies showed relatively small differences in the composition of peripheral (21) and splenic (19) mononuclear cells among wild and laboratory rats. Similarly, concentrations of circulating cytokines and chemokines were, overall, not different between wild and laboratory rats and for those that did differ (5 of 23 measured), the laboratory rats had higher cytokine concentrations than the wild rats (22). However, as for studies of wild mice, many measures of immune status were much more variable among wild rats than among laboratory rats (20, 22). Other studies found that wild rat spleens were larger (as a proportion of body mass) than those of laboratory rats, and that their splenocytes proliferated less, and their T cells did not upregulate expression of CD25 or CD134 and produced less IL-2 and TNF-α, but significantly more IL-4, in response to *in vitro* Con A stimulation (19, 20). Finally, both of these studies observed lower circulating concentrations of TNF-α in wild rats compared with laboratory rats (19, 22). For measures of humoral immunity, wild rats antibody titres (IgE, G, and M) were higher than those of laboratory rats, consistent with the likely greater antigen exposure of wild animals (12, 19). Increased exposure to infection was also indicated by the high prevalence of chronic, inflammatory lung (among 33% of animals), and kidney (among 25% of animals) disease, commonly accompanied by increased numbers of peripheral leukocytes, in wild rats that was not seen in laboratory rats (21).

Similarly, a study comparing wild and laboratory-maintained cotton rats, *Sigmodon hispidus*, also found larger spleens (both absolutely and as a proportion of total body mass) containing more cells, but lower proliferative responses to pokeweed mitogen (PWM) and Con A in wild rats compared with laboratory animals (18).

All told, these six studies of two species of rats tend to show rather subtle differences between wild and laboratory animals, which cannot easily be classified as one population being either more or less immunologically active than the other. However, as observed for wild mice, wild rats tend to have higher antibody titres but their generic (i.e., mitogen driven) spleen cell proliferative capacity and cytokine production is lower than that of laboratory animals.

A comparison of haematological parameters between wild and wild-caught, but captive, voles, *Microtus agrestis* showed that captive animals had comparatively higher erythrocyte, lymphocyte and monocyte densities, with a notable decline in lymphocyte density in both males and females when reproduction began (23).

Collectively, these 14 studies of mice, rats and voles show that wild rodents’ immune systems are generally in a highly antigen-experienced state (seen as comparatively more mature, effector lymphocyte populations and higher antibody titres) consistent with these wild animals being subject to sustained antigenic exposure. *In vitro* functional responses (to mitogens and microbial products) show a more mixed picture, with often fewer differences between wild and laboratory animals and often lower responses in wild animals. Critically, highly elevated proliferative or cytokine responses—putatively commensurate with the elevation of other aspects of the immune response—are not seen. While we could speculate about the reasons for this apparent disconnect between past immune experience and current immune function, there are currently too few studies, and these studies are too limited in scope, to allow robust conclusions to be drawn.

### Other Studies of Immune Responses of Wild Rodents

There have been other observational studies of the immune status of wild (or near wild) rodents, but with no explicit comparison to laboratory animals, which we will now review.

For wild *S. hispidus, in vitro* splenocyte proliferative responses to PWM were greater than to Con A, but both responses varied in a similar pattern across seasons, but mitogen-induced responses tended to vary inversely with spontaneous proliferation (reflecting the *in vivo* activation state of the cells) (26). In wild-caught cotton rats (or their first-generation captive offspring) the size of Peyer’s patches increased with animals’ age as did the number of large intestine lymphoid follicles (24). Experimental manipulation of animals’ diet (both wild caught, but then captive maintained, but also including some captive colony animals) affected investment in lymphoid tissue, with the total mass of Peyer’s patches being smaller in animals fed a low (4%) protein diet, compared with those fed a 16% protein diet, though Peyer’s patches were a significantly greater a proportion of body mass in low protein diet (24). In animals (wild caught and from an outbred captive colony) fed low protein diets, their spleen, thymus, and packed cell volumes were generally lower, compared with those fed the high protein diet (25). These diets also had functional effects, with low protein diet-fed animals having comparatively lower serum complement activity but higher delayed type hypersensitivity (DTH) responses (25).

There have been three immunization studies of brown rats, *R. norvegicus*. This found that antibody titres varied widely among rats immunized with a polymeric peptide, with their responsiveness linked to their MHC haplotype (27, 29). Similarly, wild rats (*R. rattus*) infected with the plague bacterium (*Yersinia pestis*) developed long-lasting IgG and IgM responses, though there was heterogeneity of responses among animals from different locations (28).

There have been a number of studies of wild voles, *Microtus* spp. A longitudinal survey of wild *M. agrestis* populations found that erythrocyte density declined in the spring, peaking in the autumn; lymphocyte density was greatest in summer and autumn, and tended to decline in older animals; neutrophil density peaked in spring, and monocytes behaved similarly, though slightly later (23). There were some sex effects, with neutrophil and erythrocyte densities being higher in males, with reproduction also affecting many measures, but often in complex ways connected with prior animal density (23).

Longitudinal studies of *M. agrestis* revealed that anaemia (as an indicator of poor body condition) predisposed individuals to monocytosis (indicative of infection), whereas the effect of lymphocyte density with regard to monocytosis varied with animal density, and that monocytosis preceded a decline in lymphocyte numbers and erythrocyte density (30).

Also in *M. agrestis*, transcriptional analysis of genes coding for cytokines (TGF-β1, IL-1β, IL-10, and IFN-γ) in response to toll-like receptor (TLR)-2 and TLR-9 agonists, and expression of transcription factors related to immunological function (FoxP3, Tbet, Gata3, and IRF5) in PHA-stimulated splenocytes, showed strong seasonal effects (31). Specifically, pro-inflammatory responses were elevated in late winter and early spring, declining thereafter, whereas anti-inflammatory responses, generally, declined as day length increased, though there was heterogeneity among individual anti-inflammatory markers (31). Markers of anti-inflammatory responses also tended to decline as animals moved from non-mating status, to mating, then gravid status, while markers of inflammation were negatively associated with body condition (32). In *M. agrestis*, longitudinal analysis of the expression of genes coding for IFN-γ, Gata3, and IL-10, showed that individuals consistently differ in their expression of IFN-γ, with some evidence showing that the other two genes were also consistently expressed differently (32). In *M. ochrogaster*, complement-dependent haemolysis and IL-2-dependent splenocyte proliferation both varied temporally, though without a clear, repeatable annual pattern (33). Females’ reproduction reduced their haemolysis responses and affected splenocyte proliferation (33). Together these studies of *Microtus* spp. have shown that a range of immunological parameters can be measured, and that these are often affected in complex ways by animals’ state and their environment, which includes demographic characteristics of the relevant populations, as well as infection.

There has also been immunological study of deer mice, *Peromyscus* spp. and field mice, *Apodemus sylvaticus*. Oral immunization of wild *P. leucopus* with a *Borrelia burgdorferi* antigen induced dose-dependent IgG responses, as well as increases in the density of peripheral neutrophils and eosinophils (34). In *P. maniculatus*, anti-Sin Nombre virus IgG titres were higher in males, compared with females, and males’ antibody titre was positively related to body mass (35). In *A. sylvaticus, in vitro* splenocyte stimulation with TLR-2 and TLR-9 agonists induced high levels of TNF-α secretion and these responses (summarized as principal components) were negatively correlated to infection with a helminth and an ectoparasite (suggestive of pathogen-induced immunomodulation), but were not related to animals’ size, mass, or life-history state (36). Primary immune responses to SRBC immunization *P. leucopus, M. pinetorum, Neotoma floridana, Onychomys leucogaster*, and *Perognathus hispidus* have been induced and were all heterogeneous among the individual animals studied (11).

These 15 diverse studies (Table 1) of other wild rodents, where there is no explicit comparison to laboratory animals, shows that immune responses differ among individuals, that effects of season and other aspects of animals’ environments are important, as too is age, though there are often no reported sex differences, though reproduction can be a significant immunological event. These studies also show that infections are important in affecting an animal’s immune status.

## Understanding the Immune Responses of Wild Rodents—A Synthesis and Questions for the Future

Taken together, a coherent picture of wild rodent immune responses begins to emerge from these 26 studies. Specifically, that the humoral, and adaptive and innate cellular compartments of their immune systems are highly active—and more so than those of laboratory animals—with this immune phenotype entirely consistent with high level and continuous antigenic challenge (especially compared with laboratory animals). But functional cellular responses of wild rodents—in particular, proliferative and cytokine responses to broad spectrum stimuli such as mitogens or TLR agonists—are lower than those observed in laboratory animals. There is therefore a disconnect between different aspects of the immune biology of wild animals when judged against the accepted paradigms derived from studies of laboratory animals. Specifically, in wild rodents, despite evidence of extensive prior and current exposure to immunological challenges (i.e., high antibody titres, highly differentiated lymphocyte populations expressing markers of recent or current activation and immune memory) their ability to respond appropriately to an additional immunological stimulus is lower than one might expect given data from laboratory animals. Although data are limited, there is some evidence that antigen-specific T cell responses of wild rodents may be similar to (or higher than) those of laboratory mice (16), such that lower-than-expected immune function may be limited to innate immune responses—but much more work is required to determine whether this is indeed true.

Notwithstanding this current unknown, an important question is: which of these situations is “normal” and which is “abnormal”? Are wild animal responses abnormally low, or are laboratory animal responses abnormally high? Perhaps the most parsimonious explanation to this paradox is that, in the face of persistent or repeated infectious challenge, high-level proliferative and cytokine responses can cause severe tissue pathology, and to avoid this, functional immune responses of wild rodents are moderated so that individuals are adequately protected from infection while avoiding immune-mediated damage. This explanation has to be understood with the context that wild animals are exposed to a very large and diverse antigenic load (key concept 3, above). By comparison, laboratory animals have a very low antigenic burden, and thus their resting level of immune activation is much lower, and they may, therefore, be able to sustain higher proliferative and cytokine responses to any individual stimulus without causing immunopathology. In support of this hypothesis, immune responses of laboratory mice share many features of the neonatal human immune response (15). If true, this idea implies that the constrained cellular immune responses of wild rodents are both mechanistically and evolutionarily appropriate. In contrast, immune responses of laboratory rodents are more typical of those of a naïve, antigen-inexperienced animal. If so, then the initial immune response of any animal (in the lab, animals entering an experiment; in the wild, newly born animals) are high, but that these then decline with continued antigenic exposure (which is the natural life course in wild animals, if they survive, but which rarely happens in laboratory experiments).

It is also important to remember that these functional immune responses are, at least in part, measures of the influence of immunoregulatory processes. There is evidence from studies in humans that immunoregulatory mechanisms accrue over the life course and in response to microbial exposure, for example, by the accumulation of regulatory T cells (37, 38), though the relevance of age-dependent effects in laboratory rodents to those of wild rodents which are typically short lived (16, 32) remains unclear. We therefore need to be mindful that while one can measure many immune parameters, these represent different, discrete aspects of the functioning immune system, ranging from frontline effector responses against a pathogen, to back-room regulatory processes. The immunoregulatory processes and the homeostatic state of wild rodents’ immune systems will be appropriate to the nature and degree of antigenic stimulation to which these animals are exposed (and so will vary among individuals within populations, among populations within species, and among species). In one sense, this is self evident—the immune system responds in a regulated way to antigenic stimulation. The immunoregulatory processes of laboratory animals may be qualitatively or quantitatively different, given the very different environments in which these animals live.

This analysis generates two important research priorities for future studies of wild rodent populations. First, to discover and quantify the totality of infections—and so antigenic exposure—to which wild animals are exposed. While we are confident in asserting that wild animals have very heavy, extensive, high-level infections, there is actually rather little substantive evidence of this. While certain infections (particularly protozoan and metazoan parasites, together with some named viruses) are quite well characterized, the microbiota and pathogen load of wild animals is poorly characterized (both qualitatively and quantitatively) (39), despite this likely being the dominant source of antigenic challenge for wild rodents. The second research priority is to understand the immunoregulatory processes of wild rodents. These processes are the proximate mechanism by which the magnitude of many effector immune responses is determined. Linking these two questions together makes it interesting to ask: what is the quantitative relationship between antigenic load, immunoregulation, and effector outputs? A quantitative understanding of the action and interaction of components of the mammalian immune system is, arguably, woefully lacking, but such quantitative understanding is likely to be necessary to understand the relationship between infection and immune responses in wild animals. Ultimately, this level of understanding is necessary to be able to make predictions about which immune responses will be induced in response to particular infections given the overall antigen load, something that is not generally possible even in laboratory animals.

Other important ideas arise from these 26 studies—that there can be strong seasonal effects on the immune system [a pattern that is commonly seen among animals more generally (40)], and that these can also be affected by aspects of animals’ state (for example, their age, reproductive status, etc.), and the infections that they have. Interpreting an immune measure of a wild animal critically depends on understanding how, and under what scenarios, the immune measures of interest change. Interpretation of single immune measures without a broader context is likely to be very difficult and unreliable. Moreover, given the immunoheterogeneity that exists among individuals, both substantial sample sizes and, ideally, longitudinal measures of immune parameters are needed to properly study the immunology of wild rodents.

The energetic costs of immune responses are well established in studies of laboratory animals and of some wild animals, particularly birds, though this has actually been rather little studied in wild rodents (7). Notably, many of the studies reviewed above have sought relationships between measures of body condition and immune responses, but very often fail to find them. As noted above, manipulation of the diet of captive wild-caught cotton rats (24, 25) affected their investment in lymphoid tissue and some immune reactions. We assume that many wild animals are resource limited, and so expect that immune responses may be submaximal. However, in truth, high levels of immune responsiveness are, in general, seen in wild rodents. There are two aspects to resolving this conundrum. The first is that resource availability will vary among individual wild animals, so that some will be well resourced, while others will be poorly resourced. The second is that animals’ immune responses will have evolved in the actual resource context of those animals, so that if animals are normally resource limited, then the optimal immune response that can be achieved under those conditions will be selected for. Showing that enhancing an animal’s diet can further increase measures of immune responses does not invalidate this view, but just shows that resource availability can alter immune responses. This, therefore, raises a third key question for future studies, which is to better understand the actual resource state of wild animals and its temporal variation, and so understand how this impacts, if at all, on animals’ immune responses.

The focus of most of these studies of wild rodents is on measures of aspects of the immune response. While this is perfectly reasonable, it is also important to bear in mind that the ultimate functional effect of these immune responses is what really matters. Understanding this is much harder, especially given the high level and heterogeneous infectious challenge that we presume wild animals are exposed to. There is substantial interest in understanding the factors that affect the zoonotic spill-over of pathogens from wild animals, including from rodents (41, 42). The immune responses that wild animals make against their infections and the effect that these have on those infections is likely to be a critical factor affecting the potential for such zoonotic spill-over. This, therefore, emphasizes the applied relevance of understanding the immune responses of wild rodents in the context of the infections that these animals harbour, especially since many of these species (*Mus, Rattus*) live commensally with humans, especially in population-dense urban areas.

## On Finding That Your Experimental Model is Wrong—Part 2

The intuition—that wild rats with their stress-filled, resource-limited lives were making very poor immune responses against the nematode *S. ratti* and so making it more common in the wild, than would be predicted from the behaviour of infections in laboratory animals—is likely wrong. From the review of the available literature (Table 1), it seems clear that wild rats’ immune systems are probably making very high-level responses to *S. ratti*, as well as to their myriad other infections. Studies of laboratory rats show that while there are a number of different effector processes acting against *S. ratti* parasites (both infective stages migrating through the host toward the gut, and adult parasites in the gut), clearance of adult stages from the host gut is achieved by an intestinal mucosal mast cell response that depends on IL-3, among other cytokines (43–45). Wild animals’ cytokine responses to antigenic stimulation are often depressed, compared with those of laboratory animals, and it is this that may underlie the apparent failure of wild rats to eliminate *S. ratti* infections. To put it formally, we can hypothesize that in wild rats infected with *S. ratti* the resultant antigenic stimulation is insufficient to generate an IL-3 response (and response of other cytokines) that is able to drive an effective mast cell response in the rat gut mucosa, with the consequence that the *S. ratti* parasites survive. Under this hypothesis the non-response could be due to ineffective (i) presentation of *S. ratti* antigen and/or (ii) subsequent induction of cytokine responses in wild rats.

## Conclusion

The immune system and its responses play a critical role in the lives of wild animals, but these responses are very often poorly understood. Immune processes are a key factor affecting the ecology of wild animals, and to fully understand the ecology of animals, these immune processes must be brought to the fore. The current small number of studies on wild rodents shows that such studies are possible, and the emerging picture of wild rodent immunology suggests some of the next research questions that the field should address.

## Author Contributions

The authors co-wrote the paper and approved it for publication.

## Conflict of Interest Statement

The authors declare that the research was conducted in the absence of any commercial or financial relationships that could be construed as a potential conflict of interest.
